# Comprehensive geriatric assessment, multifactorial interventions and nurse-led care coordination to prevent functional decline in community-dwelling older persons: protocol of a cluster randomized trial

**DOI:** 10.1186/1472-6963-12-85

**Published:** 2012-04-01

**Authors:** Jacqueline J Suijker, Bianca M Buurman, Gerben ter Riet, Marjon van Rijn, Rob J de Haan, Sophia E de Rooij, Eric P Moll van Charante

**Affiliations:** 1 Department of General Practice, Academic Medical Center, Amsterdam, The Netherlands; 2 Department of Internal Medicine and Geriatrics, Academic Medical Center, Amsterdam, The Netherlands; 3 Department of Clinical Research Unit, Academic Medical Center, Amsterdam, The Netherlands; 4 Academic Medical Center Amsterdam, Department of General Practice, Room F2-219, PO Box 22660, 1100 DD Amsterdam, The Netherlands

**Keywords:** Comprehensive geriatric assessment, Multifactorial interventions, Nurse-led care coordination, Functional decline, Community dwelling older persons

## Abstract

**Background:**

Functional decline in community-dwelling older persons is associated with the loss of independence, the need for hospital and nursing-home care and premature death. The effectiveness of multifactorial interventions in preventing functional decline remains controversial. The aim of this study is to investigate whether functional decline in community-dwelling older persons can be delayed or prevented by a comprehensive geriatric assessment, multifactorial interventions and nurse-led care coordination.

**Methods/Design:**

In a cluster randomized controlled trial, with the general practice as the unit of randomization, 1281 participants from 25 general practices will be enrolled in each condition to compare the intervention with usual care. The intervention will focus on older persons who are at increased risk for functional decline, identified by an Identification of Seniors at Risk Primary Care (ISAR-PC) score (≥ 2). These older persons will receive a comprehensive geriatric assessment, an individually tailored care and treatment plan, consisting of multifactorial, evidence-based interventions and subsequent nurse-led care coordination. The control group will receive 'care as usual' by the general practitioner (GP). The main outcome after 12 months is the level of physical functioning on the modified Katz-15 index score. The secondary outcomes are health-related quality of life, psychological and social functioning, healthcare utilization and institutionalization. Furthermore, a process evaluation and cost-effectiveness analysis will be performed.

**Discussion:**

This study will provide new knowledge regarding the effectiveness and feasibility of a comprehensive geriatric assessment, multifactorial interventions and nurse-led elderly care in general practice.

**Trial registration:**

NTR2653

**Grant:**

Unrestricted grant 'The Netherlands Organisation for Health Research and development' no 313020201

## Background

Functional decline in community-dwelling older persons is associated with the loss of independence, the need for hospital and nursing-home care and premature death [1]. Functional decline is defined as the deterioration of one or more activities of daily living (ADL) or instrumental activities of daily living (IADL) and it affects approximately 12% of community-dwelling persons aged 75 years and over yearly [2,3]. Present evidence suggests that with the increasing life expectancy, functional decline is postponed toward the oldest age (> 85 years) [4,5]. With an aging population, increasing levels of functional decline are expected to place a high burden on social and economic resources [6]. Therefore, there has been considerable focus on multifactorial interventions to maintain physical functioning and independence and to postpone disabilities in community-dwelling older persons [1]. Nevertheless, the effectiveness of multifactorial interventions, regarded as interventions relating to different aspects of care, for the prevention of functional decline remains controversial [1,7-10]. Previously, a meta-analysis reported no reduction in functional decline [8] whereas two later meta-analyses reported a reduction in functional decline only in programs that included a clinical examination [8,9,11]. A fourth meta-analysis showed a favorable yet modest reduction in functional decline, but no specific benefit for type or intensity of intervention was noted [1].

Despite controversy about their effectiveness, annual multidimensional assessments or preventive home visitation programs are part of national policies in several Western countries, including the United Kingdom and Denmark [12,13]. In the Netherlands, comprehensive guidelines for the care of community-dwelling older persons with multifactorial care needs are still lacking. In 2008, the Dutch government launched the National Program of Care for Elderly Persons to improve care and cure rates for older persons by stimulating innovative healthcare projects focused on multifactorial care.

The aim of this study, as part of the National Program, is to investigate whether functional decline in community-dwelling older persons can be delayed or prevented by a comprehensive geriatric assessment, an individually tailored care and treatment plan based on multifactorial, evidence-based interventions and nurse-led care coordination.

## Methods

### Design and setting

This cluster randomized trial is being conducted in 25 general practices (34 general practitioners (GPs)), with a total of 10,471 persons aged 70 years and over, in the northwestern region of the Netherlands. The region has both urban and rural communities, which is broadly representative of the general Dutch population. The study started on December 1, 2010 and in each general practice, the intervention will end after 12 months, with a final follow-up measurement after 24 months.

### Study population

General practices are eligible unless they already employ nurses for care coordination for community-dwelling older persons. At the start of the study on December 1, 2010, such programs were still rare. All community-dwelling persons aged 70 years and over who are registered with one of the participating general practices are selected from the electronic medical records by their GP. Persons are excluded if, according to their GP, they are terminally ill, suffer from dementia, do not understand Dutch, plan to move or spend a long time abroad or live in a nursing home. Eligible persons receive a letter with study information from their GP, along with a written informed consent form, a self-reporting questionnaire and a pre-paid envelope. They are invited to fill out the questionnaire themselves, but if they need help, an informal caregiver is allowed to provide help.

Those persons unwilling to participate are asked to select one of four prestructured reasons on a reply card: too ill, no health problems, not interested, or lack of time. Or, they can add their own comment. A postal reminder is sent after three weeks if no response is received. After six weeks, two attempts by phone are made to contact those who have failed to respond.

### Ethical approval and informed consent

All general practitioners are asked to provide written informed consent for their participation in the study. All participants are asked to provide written informed consent for data collection and participation in the study after receiving written study information.

The recruitment procedures are conducted in accordance with the Dutch Medical Research Involving Human Subjects Act and the WMA declaration of Helsinki. The study has been approved by the Medical Ethics Committee of the Academic Medical Center, University of Amsterdam, in the Netherlands (protocol ID MEC10/182).

### Randomization and blinding

**Figure 1:**
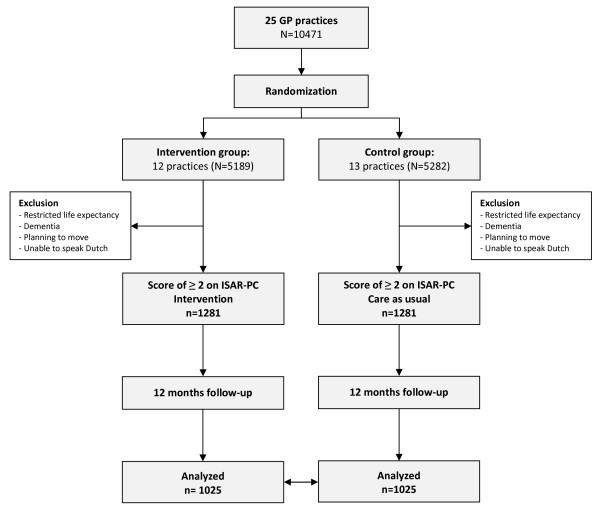
**Flowchart of the study population**.

In this cluster randomized controlled trial, with the general practice as the unit of randomization to minimize contamination [14], 25 general practices are randomly assigned to the intervention condition or the control condition (Figure 1). General practitioners who share a patient register are considered to be a single practice. A prerandomization procedure is conducted to prepare the intervention practices for the introduction of a registered nurse (RN) specialized in care for older persons, to provide instructions about the study protocol to the RNs and GPs and to plan home visits for respondents at increased risk for functional decline in the intervention practices.

Computerized randomization is performed by an independent statistician. To ensure sufficient balance between the intervention and control condition, the randomization procedure contains the following restrictions: (1) for both study arms, the total number of potential participants should not differ by more than 250 persons, (2) the number of general practices should not differ by more than five practices and (3) the absolute difference in the proportion of participants with a high socioeconomic status score should not differ by more than 20%. The first computer-randomized list generated that fulfills all criteria will be used.

With regard to the intervention, a postponed informed consent procedure has been chosen to blind all participants in both arms [15]. This procedure is applied to prevent selection bias; subjects in the control condition may be less motivated to participate if they are aware that they are assigned to a control cluster [15]. Similarly, participants in the intervention condition may influence their self-rated scores if they are aware that a higher score may lead to nurse-led care [16]. In the intervention condition, eligible participants are further informed about the procedure of the comprehensive geriatric assessment (CGA), the individually tailored care and treatment plan (CTP) based on multifactorial interventions and the nurse-led care coordination, but they are not otherwise informed that this is the intervention under study. As explained in the study information, participants in both study groups will receive written information on the complete study objectives and outcomes after termination of the study.

Blinded research assistants will conduct all physical follow-up assessments after 12 months in the intervention group.

### Sample size calculation

On the basis of a consensus within the study group, we determined the smallest meaningful clinical difference to be 0.5 points on the primary outcome measure (Katz-15) within the group of participants at increased risk of functional decline (ISAR-PC score ≥ 2). Observational data from primary care practices from a prospective cohort study (mean Katz score 2.70, SD 2.55) indicates that this would represent an effect size of 0.20. With an assumed ICC of 0.015 [17] and an expected cluster size of 100 participants per practice, the design effect would amount to 2.50 (1 + 100*0.015). Using a two-sided alpha of 0.05 and power of 80%, 1025 participants would be needed in each group, taking into account the design effect (unadjusted 410). To allow for a drop-out rate of 20% within one year, the final target sample of participants was increased to 1281 per treatment arm.

### Identification of older persons at increased risk for functional decline

The ISAR questionnaire was originally developed to identify older persons at risk for functional decline who visited the emergency department [18]. The questionnaire consists of six dichotomous, self-rated questions on the dependence of ADL and IADL, recent hospitalizations, impaired memory, visual impairment and polypharmacy. In a prospective cohort study with 790 patients, the ISAR was adapted and validated for the prediction of functional decline at 12 months in community-dwelling older persons (ISAR-Primary Care, ISAR-PC). The ISAR-PC comprises four simple, self-rated questions on the dependence of ADL and IADL, impaired memory and age (70-75 years; 75-85 years; > 85 years) that can be rated on a score card (Table 1). At a cut-off of two points, the ISAR-PC reaches an optimum, with an area under the curve (AUC) of 0.70 (95% CI 0.68-0.71) (*Unpublished data*). Therefore, all participants with a score of two points or more on the ISAR-PC, which is approximately 39%, are considered to be at increased risk for functional decline and are eligible for the intervention.

**Table 1:** Scorecard: Identification of Seniors At Risk - Primary Care (ISAR-PC)

ISAR-PC		
1. Did you need assistance for IADL on a regular basis in the last month (e.g., assistance in housekeeping, preparing meals, shopping)?	No	0.0
	Yes	2.5

2. Did you need assistance for ADL in the last 24 hours (e.g., dressing, going to the toilet)?	No	0.0
	Yes	2.0

3. Do you regularly have memory problems?	No	0.0
	Yes	2.0

4. Your age is:	74 year or younger	0.0
	Between 75 en 84 year 85	1.5
	year and older	3.0

Total score		...

Total score 0 or 1: Not at risk

Total score ≥ 2: Patient at risk for functional decline

Maximum score: 9.5 points

### Baseline assessment

The self-reporting questionnaire that is conducted at baseline comprises determinants of functional decline (e.g., comorbidities) and a minimal data set (MDS) consisting of demographic data, physical functioning, self-perceived health status, psychological and social functioning, health-related quality of life and healthcare consumption, as the study is conducted as part of the National Program [19]. The questionnaire takes between 20 and 30 minutes to complete (see Table 2 for further details).

**Table 2:** Measures used in the trial

Measures	Baseline	6 m	12 m	18 m	24 m
**Intervention group**					

**Self-reporting measures**					
Physical function Katz-15 index [33,32]	x	x	x	x	x
Health-related quality of life (EQ-6D) [34]	x	x	x	x	x
Psychological and social functioning (subscale Rand 36) [35]	x	x	x	x	x
Healthcare utilization [36]	x	x	x	x	x
Incidence of falls within 12 months	x	x	x	x	x
Evaluation of burden of caregivers (CarerQol) [37]	x	x	x	x	x
Mortality rate (GP registration)	x	x	x	x	x

**Comprehensive Geriatric Assessment**	x				

**Physical examination**					
BMI (kg/m^2^)	x		x		
Blood pressure (mmHg)	x		x		
Pulse (beats/min)	x		x		
Grip strength (kg)	x		x		
Walking speed (m/s)	x		x		

**Process evaluation**					
Patient interviews			x		
Registred nurse interviews			x		
Interviews general practitioners			x		

**Control group**					

**Self-reporting measures**					
Physical function (modified Katz ADL index score) [31,32]	x	x	x	x	x
Health-related quality of life (EQ-6D) [34]	x	x	x	x	x
Psychological and social functioning (subscale Rand 36) [35]	x	x	x	x	x
Healthcare utilization [36]	x	x	x	x	x
Incidence of falls within 12 months	x	x	x	x	x
Evaluation of burden of caregivers (CarerQol) [37]	x	x	x	x	x
Mortality rate (GP registration)	x	x	x	x	x

**Physical examination (aselect sample)**					
BMI (kg/m^2^)	x		x		
Blood pressure (mmHg)	x		x		
Pulse (beats/min)	x		x		
Grip strength (kg)	x		x		
Walking speed (m/s)	x		x		
BMI (kg/m^2^)	x		x		

### Intervention

The intervention consists of a CGA, an individually tailored CTP based on multifactorial interventions and nurse-led care coordination with components of both disease and case management. All components of the intervention are conducted by an RN and are described in the study protocol (see the web appendix, Additional file 1 for further details).

### Registered nurse specialized in care for older persons

In total, fourteen RNs are taking part in the intervention. Both before and during the trial, the RNs follow a 10-day training program in which they are educated on the content and use of the study protocol, the CGA and how to design and apply the individually tailored CTP. Much emphasis is placed on care coordination, patient empowerment and motivational interviewing. Following the training, they will attend a group refresher course every six weeks on the content of the study protocol and discuss complex cases. Each individual RN's work-up and care coordination of complex cases is critically reviewed twice.

### Comprehensive Geriatric Assessment

The CGA is conducted during the first two home visits to systematically identify geriatric conditions, problems and needs that are frequently encountered with community-dwelling older persons. The CGA focuses on the physical, psychological, functional and social domains, such as urinary incontinence, memory problems, fall risk and loneliness, respectively (Table 3). For identification of these conditions, the CGA comprises a bundle of internationally validated instruments. While some instruments are used for all participants (e.g., Mini-mental state examination (MMSE)) [20], the use of others is preceded by a positive answer on one or two screening questions (e.g., Geriatric Depression Scale-15 (GDS-15) is preceded by Geriatric Depression Scale-2 (GDS-2)) [21]. If such instruments are not available, we use commonly used items from the guidelines or literature to inquire about the presence or absence of certain problems (e.g., 'do you experience urinary incontinence?'). These questions were piloted among a small group of GPs and patients before they were incorporated into the CGA. After completing the full checklist in all four domains, a physical examination takes place that includes height, weight, blood pressure, pulse, handgrip strength and walking speed. The latter measurements make it possible to define the population along the Fried criteria for frailty [22].

**Table 3:** Content of the comprehensive geriatrc assessment (CGA)

Domain	Question (Q) or instrument (I) in CGA	Condition/disease
**Physical**		
Medication	Do you experience difficulties or side effect with medication use? Polypharmacy defined as the use or three or more different medications Medication adherence (questionnaire of Aburuz) [55]	Medication safety and side effects Polypharmacy Medication adherence
Mobility and stability	Have you fallen once or more in the past twelfth months? [56] Fear of falling (FES-I) [57] Do you experience dizziness? Fracture risk score [57,58]	Falls Fear of falling Dizziness Osteoporosis risk
Nutrition	Short Nutritional Assessment Questionnaire (SNAQ) [59] Have you been admitted to a hospital because of dehydration? Difficulties with swallowing? Do you have pain in your mouth?	Malnutrition Dehydration Swallowing disturbance Oral hygiene
Urine and fecal problems	Do you experience urinary incontinence? Do you experience fecal incontinence? Do you have a indwelling urinary catheter? Do you experience obstipation?	Urinary incontinence Feacal incontinence Indwelling urinary catheter use Obstipation
Skin	Do you have pressure ulcer(s)?	Pressure ulcer
Pain	Visual analogue scale for pain [60]	Pain
Allergy	Are you allergic?	Allergy

**Phychological**		
Cognition	Do you have memory problems? Mini- Mental State Examination (MMSE) [20]	Cognitive impairment
Delirium	Have you ever experienced a delirium? Confusement Assessment Method(CAM) [61]	Delirium
Depression	Geriatric depression Scale (GDS-2, GDS-15) [21,62]	Depression
Anxiety	Do you feel anxious?	Anxiety
Dependency	Do you smoke? Use of alcohol [63,64] Do you use benzodiazepines?	Alcohol, smoking and medication use

**Functional**		
ADL functioning	Modified Katz ADL index score [31]	ADL dependency
IADL functioning	IADL questions of Lawton and Brody [32]	IADL dependency
Mobility difficulty	Are you using a walking aid?	Mobility difficulty
Hearing	Do you experience difficulties with hearing, despite the use of a hearing aid?	Hearing impairment
Visual	Do you experience difficulties with your vision, despite the use of glasses?	Visual impairment
Sleep	Do you experience problems with sleeping? Do you use sleeping medication? If yes, how often?	Sleeping disorder

**Social**		
Loneliness	Jong Gierveld-questionnaire [38]	Loneliness
Finance	Can you manage financially?	Finance
Health related quality of live	EQ-6D [34]	Health related quality of live
Burden of caregiver	CarerQol instrument [37]	Burden of caregiver

**Physical examination**		
Body mass index	(kg/m2)	Obesity or weight loss
Blood pressure	(mmHg)	Hypertension
Pulse	(beats/min)	Pulse
Grip strength	Maximal grip strength in the dominant hand (kg) [22]	Frailty
Walking speed	Walk three meter at usual pace (seconds) [22]	Frailty

The CGA is based on previous experience from the DEFENCE study [23] by an expert panel consisting of two geriatricians, two GPs and two RNs. Four older, dedicated volunteers with experience in healthcare supported the expert panel by validating the content of the chosen geriatric conditions and problems that are evaluated in the CGA. The CGA takes about 40 to 60 minutes and its feasibility was tested during a pilot phase among 20 randomly chosen older persons aged 70 years and over in two general practices.

### Prioritization of conditions for individually tailored treatment and/or care

After the CGA, participants are asked if they recognize the identified geriatric conditions and potentially unmet needs, if they would like any help with or treatment for them and in case of multiple issues, with which set of problems they would prefer to start.

During the second home visit, further diagnostic assessments will follow for the identified problems/conditions based on standardized protocols (*see 'uniformity in diagnostics and interventions')*. Subsequently, the diagnostic yield of both home visits will be discussed with the GP to develop an individually tailored CTP that is prestructured within the same protocols and based on multifactorial interventions. After this meeting, a third home visit will be used to discuss the CTP with the participants and their caregivers. Potential discrepancies between the priorities of the patients, RNs and GPs will be addressed to find a consensus on the CTP.

### Collaboration with the General Practitioner

During the intervention, the RN will work in close collaboration with the participant's GP. They will meet weekly, at a fixed time, to discuss the CGA and finalize each individually tailored CTP. Subsequently, the RN will evaluate the outcomes and changes of the participant's CTP and the need for continuation of care coordination. The GP remains formally responsible for all care and treatment that participants will receive during the intervention.

### Home visits

After the CTP is discussed with the participant, the following themes will be addressed during the subsequent home visits (up to seven in total):

(a) the CTP and the initiated interventions are evaluated and adjusted if necessary. A summary of the CTP will be saved in the GP's electronic medical records (EMR);

(b) prioritizing the identified geriatric conditions. During the intervention, geriatric conditions may change, as may participants' prioritization of them;

(c) social functioning and participation;

(d) the burden and needs of a participant's caregiver;

(e) the participant's needs and expectations. The RN enhances empowerment of the participants and caregivers by providing or facilitating psychoeducation on the identified geriatric conditions [24].

The home visits required are flexible in number and timing but are aimed to be in a range between three and eight home visits. Every six to eight weeks, or at shorter intervals if necessary, the RN will visit the participant and evaluate the CTP. The last home visit is planned 12 months after the start of the intervention. Attrition of vulnerable older persons is frequently encountered in trials conducted in this population [25]. To minimize the burden, most of the interventions will take place within the home setting and an informal caregiver is invited to enhance the participants' adherence to the intervention. To build a strong and trusting relationship between the RN and the participant and family, healthcare coordination is performed by only one or two RNs per participant.

### Nurse-led care coordination and protocol

Nurse-led care coordination consists of elements of disease and case management, self-management and caregiver support, which are derived from several chronic care models [26-29] and adapted for the Dutch healthcare system. The themes in all four domains of the CGA are potential targets for care coordination and are embedded in the study protocol (web table 3). The RN works in close collaboration with the GP and maintains contact with other healthcare professionals (e.g., occupational therapists, physiotherapists, older persons' welfare consultants, etc.) and the participant's caregiver. Many older persons with several chronic conditions are already in the care of multiple different healthcare professionals at the same time. A comprehensive inventory will be made of all collaborating healthcare professionals and the overall care coordination that may already be in place or is (still) needed. If needed, the RNs will start or expand care coordination.

### Uniformity in diagnostic work-up and interventions

To create uniformity in screening, diagnostic assessments and interventions, a toolkit has been constructed that underpins the individually tailored CTP [http://www.effectieveouderenzorg.nl, *in Dutch*]. The toolkit has been created for the purpose of the present trial, based on previous experience from the DEFENCE study, and consists of standardized protocols for all geriatric conditions that follow international guidelines and are all evidence based or based on current best practices [23]. The protocols share a common structure: a goal to achieve while intervening in a geriatric condition, step-wise action plan, background information (prevalence, risk factors), screening for conditions (appropriate question(s) or validated instruments), indications for further diagnostic work-up, evidence-based interventions, financing of care and advice for participants and an aim of patient empowerment.

For the design and development of the protocols in the toolkit, the expert panel was extended with two occupational therapists, a physiotherapist experienced in geriatric physiotherapy, a nurse specialized in geriatric nursing and an elderly welfare consultant. The extended expert panel worked in close collaboration with four older volunteers with experience in healthcare who will also monitor the study on behalf of the older persons participating in the National Program. Each protocol was written by two members of the expert panel and was accepted to be externally reviewed if at least two other members agreed with the content. All of the protocols were critically reviewed by an external multidisciplinary expert panel, consisting of two geriatricians and four GPs, who paid specific attention to the protocol's correspondence to current guidelines and latest evidence and to its feasibility in general practice. The expert panel met ten times in total.

### Control group

Participants registered with general practices that are randomized to the control group will receive unrestricted care as usual according to the current guidelines for Dutch general practice [30]. This care may vary from on-demand care by GPs to regular home care involvement via the GP.

### Outcomes and measurements

All of the participants in both conditions will receive similar postal questionnaires at baseline and after six, 12, 18 and 24 months.

#### Primary outcome

The primary outcome measure is the self-reported level of physical functioning at 12 months, measured with the modified Katz-15 index score [31,32]. This index measures six basic ADL items (bathing, dressing, toileting, eating, continence and transfer) and nine IADL items (housekeeping, meal preparation, shopping, combing hair, telephone use, transportation, medications use, budgeting and walking). Each item is scored 0 (independent) or 1 (dependent), with an overall score ranging from zero to 15; a higher score indicates a higher dependence in ADL and IADL [31-33]. At all time points, the questionnaire will be filled out by the same person (patient or informal caregiver).

#### Secondary outcomes

The secondary outcome measures are also based on the results of the self-reported questionnaires (at 6, 12, 18 and 24 months) in both groups (Table 3):

1. health-related quality of life (EQ-6D); [34]

2. psychological and social functioning (subscale Rand-36); [35]

3. healthcare utilization (institutionalization, hospitalization and/or visits to the emergency department of the hospital, care provided by a GP during and after hours, other professional care and informal care); [36]

4. incidence of falls within 12 months after the start of the study;

5. evaluation of the provision of care by caregivers and burden of caregivers (CarerQol); [37]

6. overall mortality rate (GP registration).

#### Tertiary outcomes

Tertiary outcomes are restricted to the intervention group and include changes from baseline in the following:

1. characteristics of frailty (unintentional weight loss (kg), weakness (grip strength (kg)), low endurance (self-reported), slowness (walking speed (m/s)), level of physical activity (self-reported)); [22]

2. blood pressure (mmHg);

3. loneliness (De Jong Gierveld scale of loneliness) [38].

### Other measurements

At baseline and after 12 months, a random sample of respondents with an ISAR-PC score ≥ 2 in the control group will receive physical measurements similar to the intervention group to facilitate a secondary analysis on quantitative, objectives scores (e.g., walking speed).

### Loss to follow up

Subjects declining (further) participation will be asked permission for a short telephone interview after 12 months to assess the modified Katz-15 index score and health-related quality of life (EQ-6D). Institutionalization and death will be derived from the GPs' electronic information system.

### Process evaluation

The study includes a process evaluation on the level of the participant, the RN and the GP. The qualitative data of semi-structured interviews with participants, RNs and GPs by two researchers, JS and MvJ, will be analyzed to evaluate the feasibility and the practicability of the intervention and to identify factors that could facilitate or inhibit the future implementation of the care program. The process indicators are formulated by the study group, based on a consensus on all elements of the intervention, and the adherence to them is evaluated by measuring their pass rates. The process indicators include the number of older persons at increased risk for functional decline, number of completed CGAs, protocols used for the identified geriatric conditions, problems incorporated in the CTP and sessions on CGA and CTP, organized by nurse and GP. The process evaluation will be monitored by older volunteers with experience in healthcare.

### Data analysis

All of the analyses will be based on an intention-to-treat principle and will be blinded for the allocation group until all analyses have been completed. The baseline data will be summarized using descriptive statistics. The main analysis focuses on the effectiveness of the interventions on the functioning of older persons through the modified Katz-15. Participants with a similar risk profile (based on ISAR-PC) in the two groups will be compared. The difference between the two groups will be evaluated using a multilevel analysis, as the modified Katz-15 index scores are expected to cluster within GPs and RNs. We will adjust the effect size for baseline imbalances caused by age, sex, socioeconomic status, baseline ADL and IADL functioning and cognitive functioning. The same multilevel approach will be used for all secondary outcomes. Survival data (e.g., institutionalization, mortality) will be additionally analyzed using Cox regression. In all of the analyses, statistical uncertainties will be quantified with corresponding 95% confidence intervals.

## Discussion

### Main results

This protocol for a cluster randomized controlled trial is designed to prevent functional decline in community-dwelling older persons through the provision of a comprehensive geriatric assessment, an individually tailored care and treatment plan based on multifactorial interventions and nurse-led care coordination.

### Other studies

Previous meta-analyses on the effectiveness of multifactorial interventions in preventing functional decline among community-dwelling older persons have yielded inconsistent results. It has been suggested that the differences in effectiveness may be explained in part by the selection of the study population and setting, the nature of the intervention(s), and adherence [1,7-10].

First, the selection of the populations is based on the general population or on high-risk populations according to age [39,40], a combination of risk factors [26,27,41,42] including frailty [43-47], self-reported poor health [48,49], or functional decline [41,42,50]. Ferrucci et al. claimed that preventive interventions should primarily target high-risk persons rather than the general population because high-risk persons are the most likely to benefit [25]. With regard to age, two meta-analyses reported that multifactorial interventions reduced mortality in a younger population (mean age < 80 years) rather than an older study population [9,10]. However, neither the effects of high risk nor age were confirmed by the recent meta-analysis by Beswick et al [1].

Three recent Dutch studies on preventive interventions for frail older people found no effect on functional status [47-49], or the positive effect was not persistent [47]. These results can be explained in part by the selection of a population that was already too frail to benefit from preventive interventions. Pre-frail elderly persons might benefit more from preventive interventions, based on the hypothesis of potential reversibility in an earlier stage of functional decline. Similarly, targeting frailty, defined from a multifactorial perspective or self-reported poor health, seems to be a less sensitive selection, leaving less room for prevention of functional decline.

Second, the features of preventive interventions that are associated with prevention of functional decline are multidimensional (i.e., geriatric assessment including physical examination, long-term follow up [9,10,51], interdisciplinary teamwork, and care coordination) [26,27]. However, no specific benefit based on the type or intensity of intervention was noted in the recent meta-analysis by Beswick et al., who suggested to focus on individually tailored interventions and care [1].

Third, others have suggested that inconsistent results on the benefit of preventive interventions may be explained in part by a large variability in adherence to the intervention or the competence profiles of the nurses taking part in the study [48,49].

### Strengths and limitations

This study has several strengths. First, the internal validity is good firm because it is a cluster RCT with postponed informed consent, limiting the risk of response bias in both groups. Second, we aim, in part, to target a younger population (70-75 yrs), in which functional decline is not yet manifested or is still emerging, with a greater preventive potential than older or more frail groups. Similarly, a sensitive selection within an older population (> 75 yrs) at increased risk of functional decline may represent a group with broader opportunities for preservation of independent functioning. To identify older persons who are at increased risk for functional decline, the ISAR-PC screening test is used. Other screening instruments for functional decline were considered less appropriate because they are not validated for the Dutch population and are insufficiently adapted for prediction over time[52-54].

Third, the intervention encompasses a *systematic *comprehensive geriatric assessment, with multiple home visits, an individually tailored CTP, based on multifactorial, evidence-based interventions and nurse-led collaborative care coordination. All of these features are associated with prevention of functional decline and are combined in the current intervention. Fourth, the study includes a process evaluation to evaluate the feasibility and the practicability of the intervention, the adherence to the intervention, the competence of the RNs and any factors that could facilitate or inhibit future implementation.

Finally, the overall intervention is designed and monitored in collaboration with older volunteers to increase both internal and external validity.

The study also has some limitations. First, the nature of functional decline of individual community-dwelling older persons is dynamic, which makes it difficult to predict functional decline [3].

Second, in this study, persons with dementia are excluded. Although they are clearly at high risk for functional decline, a full range of services for dementia patients is already established in the region, including case management.

Third, the window of opportunity for preventing or delaying functional decline appears to be small. In a recent meta-analysis, a standard mean difference (SMD) of 0.08 was described, which equated to about a half-point improvement in the applied 20-point score [1]. Nevertheless, in view of national plans toward a more proactive care for community-dwelling older people, a large RCT in the Netherlands is still needed to explore the effectiveness of a proactive multifactorial intervention on the prevention of functional decline in older persons who are at increased risk for functional decline. Overall, the improvement on functional status in the Netherlands might be more substantial, as the Dutch healthcare system still lacks comprehensive assessments for older persons.

In summary, this preventive intervention, based on a comprehensive geriatric assessment, an individually tailored CTP of multifactorial interventions and subsequent nurse-led care coordination, has the potential to effectively prevent functional decline in community-dwelling older persons and promote self efficacy. This study is being conducted as part of the Dutch National Care for the Elderly Program. The current study will also provide information on the feasibility of innovative quality programs of care to preserve independent living in community-dwelling older persons.

## Abbreviations

ISAR-PC: Identification of Seniors at Risk Primary Care; GP: general practitioner; ADL: activities of daily living; IADL: Instrumental Activities of Daily living; RN: registered nurse; CGA: comprehensive geriatric assessment; CTP: care and treatment plan; AUC: area under the curve; SD: standard deviation; MDS: Minimal Data Set; MMSE: Mini-mental state examination; GDS: Geriatric depression scale; DEFENCE: Develop strategies Enabling Frail Elders New Complications to Evade; EMR: electronic medical records; EQ-6D: Six-Dimensional EuroQol instrument; SMD: standard mean difference

## Competing interests

The authors declare that they have no competing interests.

## Authors' contributions

JS drafted the manuscript. JS and EMC wrote the protocol for the Medical Ethics Committee. BB, MvJ, RH, GtR, SR en EMC critically reviewed the manuscript and protocol for the Medical Ethics Committee. SR drafted the research proposal. BB, RH and EMC reviewed the research proposal that was sent to the funding organization. RH was involved in the methodological construct of the study. All authors read and approved the final version of the manuscript

## Pre-publication history

The pre-publication history for this paper can be accessed here:

http://www.biomedcentral.com/1472-6963/12/85/prepub

## Supplementary Material

Additional file 1 **Web-appendix 1**. The intervention study protocol. Click here for file
