# Supplementary material for: Comprehensive geriatric assessment, multifactorial interventions and nurse-led care coordination to prevent functional decline in community-dwelling older persons: protocol of a cluster randomized trial
Source: BMC Health Serv Res. 2012 Apr 1;12:85. doi: 10.1186/1472-6963-12-85 (PMC3374886; doi:10.1186/1472-6963-12-85)
Supplement: Additional file 1 — Web-appendix 1. The intervention study protocol. [file 1472-6963-12-85-S1.DOC]

**Web-appendix 1; the intervention study** protocol

|  | **content** | **timing** | **length** (minutes) |
| --- | --- | --- | --- |
| **Home visit 1** | CGA and physical examination | week 1 | 60 |
| **Home visit 2** | Further diagnostic assessment based on standardized protocols | week 2 | 20-40 |
| **Review with GP** | Discussion of the CGA, physical examination and the CTP | week 3 | 20 |
| **Telephone contact** | The participant is informed about the CTP. Interventions are started for urgent conditions. | week 4 | 5 |
| **Home visit 3** | The CTP is discussed with the participants. Started interventions are evaluated. New interventions are started. | week 7 | 20 |
| **Home visit 4** | Social functioning and participation. Main topics of each home visit | week 16 | 20 |
| **Home visit 5** | The burden and needs of a participant’s caregiver.  Main topics of each home visit. | week 24 | 20-30 |
| **Home visit 6** | Main topics of each home visit. | week 32 | 20 |
| **Home visit 7** | Main topics of each home visit. Discussion of care after intervention. | week 40 | 20 |
| **Home visit 8** | Follow-up assessment by blinded research nurse | week 52 | 30 |

**Home visit 1**

- The systematic CGA and the physical examination are conducted
- All identified geriatric conditions and problems are summarized
- Participants are asked if they recognize the identified geriatric conditions and unmet needs and if they wish any help with or treatment for them.
- The conditions are prioritized from 1 (the most important condition) to 10 (the less important condition).
- The CTP will start with the condition with the highest priority. The other conditions may follow during the follow-up of the intervention.

**Home visit 2**

- - Diagnostic assessments based on standardized protocols are conducted for the identified conditions the participant wishes any help with.
  - If the following conditions are identified: falls, polypharmacy, pain, depression or incontinence then diagnostic assessment of these conditions have priority because they are urgent or treatment takes a long time.
  - Maximal 4-5 protocols are conducted in the second home visit. If the participant has more identified conditions then diagnostic assessment is conducted in the fourth home visit.
  - For each identified condition the participant wishes any help with a goal of treatment is specified.
  - Inform the participant about possible interventions for the conditions he whishes any help with.
  - Inform the participant about the discussion with the GP and the finalizing of the CTP. If necessary interventions can start after agreement of the CTP in a telephone contact.

**Review with GP**

- All identified conditions including further diagnostic assessments are discussed with the GP.
- The GP reviews the participant’s prioritizing of the identified conditions.
- If the participant did not give priority to a certain condition and the GP and CHN consider the condition relevant to treat, the participant will be informed about why the GP and CHN advice to have a certain condition treated.
- The CHN creates the CTP based on further diagnostics and interventions from the protocols.
- The GP and CHN finalize each CTP for:
  - The goal of treatment
  - the most suitable intervention
  - The health care professional performing the intervention
- The interval of evaluation of the CTP is determined

**Telephone contact**

- The participant is informed about the main points of the CTP.
- If necessary, interventions can be started for urgent conditions, such as falls, polypharmacy, pain, depression or incontinence.
- In case of referral to other health care professionals, they are informed by the CHN. The participants themselves make an appointment with other health care professionals.
- An appointment is made for the next home visit to discuss the CTP thoroughly and to evaluate the started intervention

**Home visit 3**

- The CTP including the priority for each condition is thoroughly discussed with the participants
- If the CTP is not complete, further diagnostic assessments based on the protocols are conducted
- Started interventions are evaluated, taking into consideration successful components and components that need adjustment
- New interventions are started
- Main topics for each home visit are discussed
  - Participants needs and expectation
  - Care coordination including regular health care visits

**Home visit 4**

- Main topics for each home visit are discussed
  - participants needs and expectation
  - care coordination including regular health care visits
  - prioritizing of identified problems, are there identified problem that gained in urgency
  - evaluation of started interventions
- Social functioning and participation appears to be very important for community dwelling elderly. In an open conversation the social functioning and participation is discussed with the participant. The conversation includes the following topics:
  - The participant’s social networks consist of?
  - Is the participant convenient with its social network?
  - Which activities does the participant undertake?
  - What would the participant like to undertake; considering the possibilities?
  - Did he used to have a hobby in the past?
  - Are the any impediments to undertake social activities?
  - Is the participant aware of the possibilities in his community?
  - Are there problems in transport that obstruct the participant to undertake social activities?
  - Are there financial problems that obstruct the participant to undertake social activities?
- Inform the participant about the possibilities to undertake social activities, depending on the result of the conversation.

**Home visit 5**

- Main topics for each home visit are discussed
  - participants needs and expectation
  - care coordination including regular health care visits
  - prioritizing of identified problems; are there identified problems who gained in urgency
  - evaluation of started interventions
- The participant’s caregiver is invited to discuss his burden and needs
- Further diagnostic assessment based on the protocol for caregivers burden is conducted
- Discuss possible interventions with the participant and its caregivers, depending on the result of the conversation
- Discuss what the caregivers can do himself to decrease the burden of the caregiver.
- Discuss if and when evaluation of the caregivers is needed and make an appointment.

**Home visit 6**

- Main topics for each home visit are discussed
  - participant’s needs and expectation
  - care coordination including regular health care visits
  - prioritizing of identified problems; are there identified problems who gained in urgency
  - evaluation of started interventions

**Home visit 7**

- Main topics for each home visit are discussed
  - participant’s needs and expectation
  - care coordination including regular health care visits
  - prioritizing of identified problems; are there identified problem who gained in urgency
  - evaluation of started interventions
- Continuation of care after the intervention is discussed.
  - does the participant desire continuation of health care coordination
  - are there any interventions that need continuation
- Inform the participant about the Follow-up assessment by a blinded research nurse

**Home visit 8**

- Follow-up assessment by a blinded research nurse
